# Identification of a factor that accelerates substrate release from SRP

**DOI:** 10.1126/science.adp0787

**Published:** 2024-11-28

**Authors:** Huping Wang, Ramanujan S. Hegde

**Affiliations:** 1 MRC Laboratory of Molecular Biology, Cambridge, UK

## Abstract

The eukaryotic signal recognition particle (SRP) co-translationally recognizes the first hydrophobic segment of nascent secretory and membrane proteins and delivers them to a receptor at the endoplasmic reticulum (ER). How substrates are released from SRP at the ER to subsequently access translocation factors is not well understood. Here, we found that TMEM208 can engage the substrate binding domain of SRP to accelerate release of its bound cargo. Without TMEM208, slow cargo release resulted in excessive synthesis of downstream polypeptide before engaging translocation factors. Delayed access to translocation machinery caused progressive loss of insertion competence, particularly for multipass membrane proteins, resulting in their impaired biogenesis. Thus, TMEM208 facilitates prompt cargo handover from the targeting to translocation machinery to minimize biogenesis errors and maintain protein homeostasis.

Secretory and membrane protein biogenesis is divided into two stages: targeting and translocation (1–3). In eukaryotes, nearly all of these proteins are targeted co-translationally to the endoplasmic reticulum (ER) (4). Targeting begins when a transmembrane domain (TMD) or cleavable signal peptide emerges from the ribosome and is recognized by the signal recognition particle (SRP) (2). Successful recognition culminates in the slotting of a hydrophobic TMD or SP into a deep, lid-covered hydrophobic groove in the M domain of SRP54, a subunit of SRP (5, 6). The SRP-ribosome complex is then delivered to the SRP receptor (SR) at the ER membrane via an interaction between the GTPase domains of SRP54 and the alpha subunit of SR (7). The SRP-SR GTPase complex re-locates away from its initial position near the ribosome exit tunnel, freeing most of this area for eventual handover to translocation factors (8, 9).

The steps downstream of this ‘pre-handover’ state are not understood, but are critical for the nascent chain to initiate translocation across or into the lipid bilayer. To minimise futile cycles of targeting, substrates must release from SRP54 and engage translocation factors before GTP hydrolysis by the SRP-SR complex triggers its disassembly (10). Furthermore, prompt handover is crucial for translocation because many proteins progressively lose translocation competence as increasing amounts downstream polypeptide emerge from the ribosome (11, 12). Yet, structural analyses show that the substrate is stably buried in a lidded hydrophobic groove (5, 6). Whereas this mode of association helps minimize premature dissociation during membrane targeting, it poses a challenge to the handover step. How the nascent chain is unloaded from SRP54’s M domain specifically and quickly at the ER membrane is not known.

It has long been thought that cargo unloading is stimulated by an ER membrane factor because SR alone cannot trigger efficient substrate release from SRP (13). Although correlative data initially implicated the Sec61 translocation channel (13), SRP substrates can be unloaded and inserted into Sec61-depleted membranes (14) and translocation of most substrates is inefficient with only SR and Sec61 (15–17). Thus, the ER seems to contains some factor(s) involved in cargo unloading from SRP (13), but its identity is unknown. By studying membrane protein biogenesis, we now show that TMEM208 is a previously unknown factor in the SRP pathway that accelerates substrate dissociation from SRP at the ER.

## TMEM208 facilitates biogenesis of multipass membrane proteins

Exploiting the recent discovery of several protein complexes involved in multipass membrane protein biogenesis (14, 18–22), we searched the cancer dependency map (23) and co-essentiality atlas (24) for co-clustering genes of unknown molecular function that might also participate in this process. TMEM208, a four-TMD ER membrane protein, uniquely and tightly clustered with multiple subunits of the ER membrane protein complex (EMC) and the multipass translocon (MPT) (fig. S1). TMEM208’s ER-localization, wide conservation, role in TRPC6 channel biogenesis (25), and links to protein targeting in yeast (via its homolog SND2) (26) and in mammalian cells (27) led us to investigate its molecular function.

**Fig. 1:**
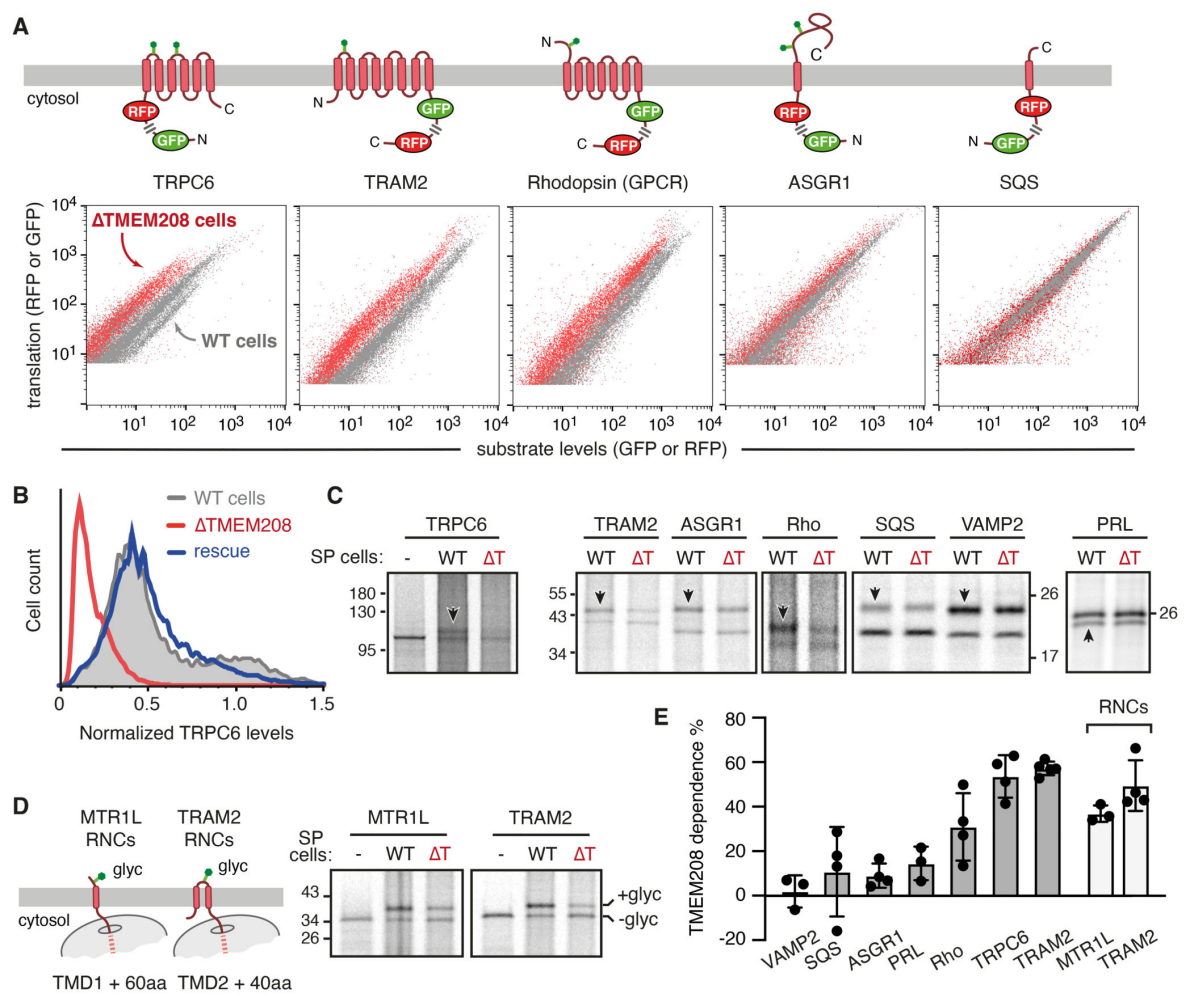
TMEM208 deletion impairs membrane protein biogenesis. (A) Dual-color fluorescent reporters of the indicated membrane proteins (top) were analyzed by flow cytometry (bottom) in either wild type (WT) or TMEM208 knockout (ΔTMEM208) HEK293 cells. Each construct contains a translation control (either GFP or RFP) separated by a ribosome-skipping 2A peptide from the membrane protein reporter tagged with either RFP or GFP, respectively. A leftward shift on the x-axis indicates reporter destabilisation relative to the translation control. (B) Histogram of the dual-color TRPC6 reporter analysed by flow cytometry in the indicated cell lines as in panel A. TRPC6 levels were normalized to the internal translation control. Rescue indicates re-introduction of untagged wild type TMEM208 into ΔTMEM208 cells by transient transfection. (C) The indicated membrane proteins were translated in reticulocyte lysate with ^35^S-methionine and wild type (WT) or TMEM208 knockout (ΔT) HEK293 semi-permeabilized cells (SP cells). Downward and upward arrows indicate the glycosylated or signal-cleaved product, respectively, indicative of translocation. (D) In vitro translation as in panel C but with truncated transcripts coding for the N-terminal regions of the indicated proteins. The diagram to the left indicates the truncated ribosome-nascent chain (RNC) length being analyzed and site of glycosylation upon successful membrane insertion. The glycosylated (+glyc) and non-glycosylated (-glyc) products are indicated. (E) Plot of TMEM208 dependence (mean ± SD; *N* of 3 to 5), defined as the percent decrease in translocation seen in ΔT relative to WT SP cells measured by assays as in panels C and D.

Consistent with the co-essentiality data, analysis of 17 fluorescent-protein-tagged reporters of multipass membrane proteins revealed a biogenesis defect for more than half of them in TMEM208 knockout (ΔTMEM208) cells (fig. S2; Fig. 1A). This included TRPC6 as seen before (25), several GPCRs, an acyl transferase (SOAT1), TRAM2, and to a lesser extent, a solute carrier (SLC12A4). Although single-pass and secretory proteins were mostly unaffected in ΔTMEM208 cells, small effects were nonetheless detectable for CYP4V2, ASGR1, prion protein (PrP) and prolactin (PRL). Two post-translationally inserted tail anchored (TA) membrane proteins [VAMP2 and SQS, whose insertion relies on the GET insertase and EMC, respectively (28)] were not affected. Cells with the lowest expression levels of reporter showed the same effects as cells with the highest expression levels (fig. S3).

The levels of the major translocation factors, particularly EMC and MPT components with which TMEM208 co-clusters genetically (fig. S1), were unaffected in ΔTMEM208 cells (fig. S4A). This is consistent with earlier proteomics analyses (29), and argues against an indirect effect on protein biogenesis. The ΔTMEM208 phenotype for the TRPC6 reporter could be rescued by exogenous TMEM208 expression (Fig. 1B). ΔTMEM208 cells showed a clear growth defect [as seen in most cancer cell lines (23)] (fig. S4B), consistent with a broad but partial and substrate-specific impairment of protein biogenesis in the secretory pathway.

## TMEM208 impacts an early step of co-translational translocation

To understand the molecular function of TMEM208, we turned to in vitro studies. We found that the substrate-specific biogenesis defects for several strongly impacted proteins from the cell-based assays (e.g., TRAM2, Rhodopsin, and TRPC6) could be recapitulated using in vitro translocation assays (monitored by glycosylation) with semi-permeabilized (SP) cells (Fig. 1C-E). Proteins whose biogenesis in cells were modestly or not affected (ASGR1, PRL, SQS, and VAMP2) were minimally or not impaired in their translocation in vitro.

Because the glycosylation sites in several of the affected proteins are near the N-terminus, we suspected an early biogenesis defect. In support of this idea, early biogenesis intermediates of multipass substrates in either topology (MTR1L and TRAM2) already displayed deficient translocation in TMEM208-lacking SP cells when analyzed at the point of initial insertion (Fig. 1D, E). This suggested that TMEM208-dependence is influenced by the targeting element of a substrate responsible for these early events. However, no obvious correlation could be seen between TMEM208-dependence and targeting sequence topology, hydrophobicity, or position (table S1), indicating a complex multi-parameter relationship (discussed later).

In contrast to these co-translationally translocated proteins, none of five post-translationally inserted TA proteins showed any appreciable difference in direct comparisons of WT versus ΔTMEM208 SP cells (Fig. 1C; fig. S5A). This contrasts with an earlier suggestion, based primarily on proteomics of cells treated with TMEM208 siRNAs (29), that TMEM208 influences biogenesis of some TA proteins. To reconcile this issue, we reconstituted ER microsomes from ΔTMEM208 cells with in vitro translated TMEM208 [which separate experiments showed was functional in restoring translocation to a TMEM208-dependent substrate (fig. S5B)]. This strategy ensures that the presence or absence of TMEM208 is the sole difference, with no compensatory or indirect effects. In this rigorous comparison, insertion of TA proteins was completely unaffected (fig. S5C). Thus, TMEM208 is needed for a subset of targeting sequences to initiate an early step of co-translational translocation.

## TMEM208 accelerates substrate release from SRP54

**Fig. 2:**
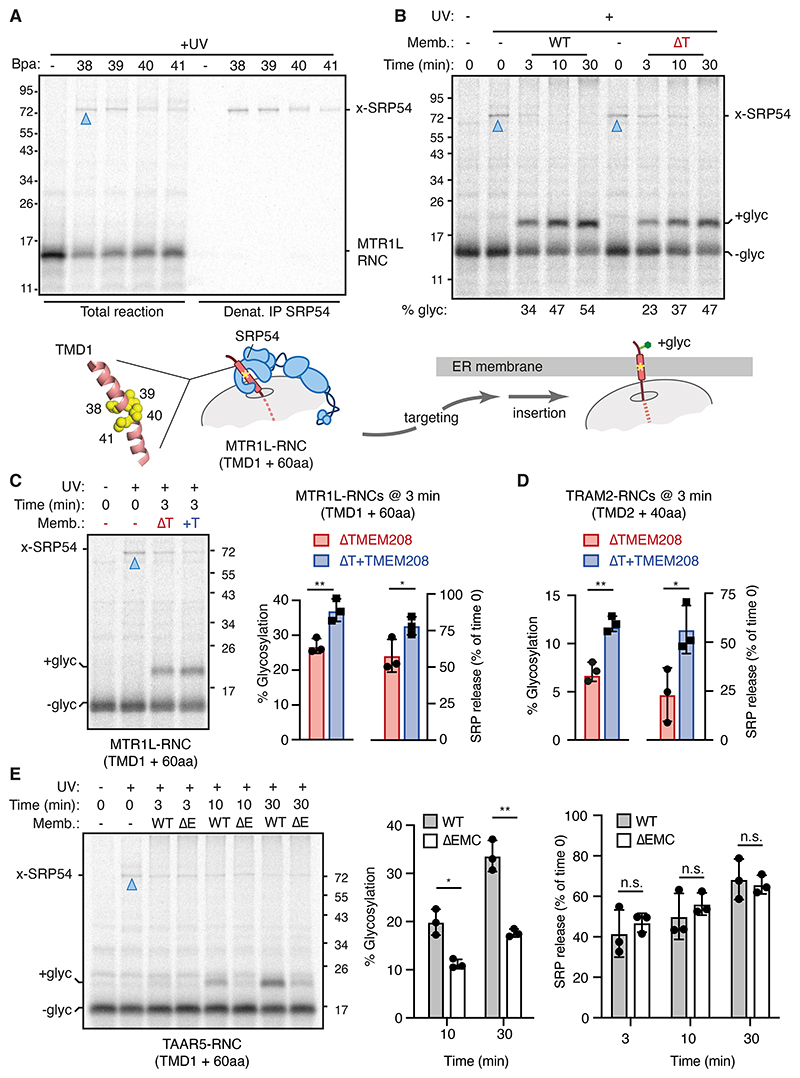
TMEM208 facilitates substrate release from SRP. (A) Ribosome-nascent chains (RNCs) encoding the N-terminal region of MTR1L were produced by in vitro translation with ^35^S-methionine and the UV-activatable crosslinking amino acid Bpa installed by amber suppression at one of four sequential positions within the first TMD (diagram at bottom). After UV irradiation, the samples were digested with RNase A to remove tRNA, denatured and analyzed by SDS-PAGE directly or after denaturing immunoprecipitation (IP) with anti-SRP54. A control construct lacking an amber codon for Bpa incorporation served as a negative control. Blue triangle indicates the major crosslink, identified by IP to be SRP54 (x-SRP54). (B) ^35^S-methionine labelled MTR1L RNCs as in panel A with Bpa at position 39 were incubated with microsomes from wild type (WT) or TMEM208 KO (ΔT) cells for the indicated times before UV irradiation. The SRP54 crosslink (x-SRP54) is lost concomitant with substrate insertion, reactions that occur slower with ΔTMEM208 microsomes. (C) Assay as in panel B at the 3 min time point using microsomes from ΔT cells without or with TMEM208 reconstitution (+T) by in vitro translation. Substrate glycosylation and percent release from SRP54 (relative to time 0) were quantified and plotted (mean ± SD, *N*=3). * p < 0.05 and ** p < 0.01 by paired Student's *t*-test. (D) Quantification of glycosylation and release from SRP54 as in panel C of ^35^S-methionine labelled TRAM2 RNCs (as in Fig. 1D) with Bpa at position 28. (E) ^35^S-methionine labelled TAAR5 RNCs with Bpa at position 48 were assayed and quantified as in panels B and C using microsomes from WT or EMC6 KO (ΔE) cells for the indicated times before UV irradiation.

Site-specific photo-crosslinking showed that cytosolic ribosome-nascent chain (RNC) complexes of MTR1L, a TMEM208-dependent substrate in cells and in vitro, engages SRP54 in the cytosol (Fig. 2A). In this experiment, amber suppression was used to install 4-benzoyl-l-phenylalanine (Bpa), a UV-activated photo-crosslinker, at each of four sequential crosslinking positions around MTR1L’s first TMD helix used for its targeting. Each of these sites crosslinked prominently to SRP54, with no appreciable other adducts from any position. This matches expectations for a TMD encased in a lidded groove in SRP54’s M domain (5, 6). Engagement of SRP is consistent with the proposed cooperation between TMEM208 and SR during biogenesis of mammalian GPI anchored proteins (27).

Consistent with a functional role for SRP, efficient TMEM208-dependent membrane insertion of MTR1L RNCs was GTP-dependent (fig. S6A). Photocrosslinking between the targeting sequence and SRP54 was seen for RNCs from seven other TMEM208-dependent substrates tested in this assay (fig. S6B, C). In each case, crosslinking efficiency was comparable to well established SRP substrates PRL and ASGR1. Furthermore, mild trypsin treatment of ER microsomes, which liberates SRP receptor (SR) from the membrane (30), sharply reduced translocation of all TMEM208-dependent substrates as seen for established SRP pathway proteins PRL and ASGR1 (fig. S6D). This loss in activity could be partially restored to similar levels for all of these substrates by including purified SR in the translocation reaction. Thus, TMEM208-dependent substrates physically engage SRP54 and functionally use the SRP pathway.

To identify the step at which TMEM208 is needed for translocation, we monitored the fate of MTR1L RNCs upon addition of ER membranes from wild type (WT) or ΔTMEM208 cells. In this multi-turnover experiment in complete cytosol and isolated ER, substrate release from SRP (as monitored by crosslinking) and subsequent insertion (as monitored by glycosylation) was slower in the absence of TMEM208 (Fig. 2B). The difference, most evident at 3 min of incubation, almost completely normalized by 30 min, at which time SRP had released equally well and overall insertion was almost the same.

This effect could be assigned solely to TMEM208, and not some indirect consequence of its loss, because ΔTMEM208 ER microsomes reconstituted with in vitro translated TMEM208 enhanced substrate release from SRP and insertion at 3 min (Fig. 2C). A similar effect was seen with TRAM2 RNCs, another TMEM208-dependent substrate of the opposite topology (Fig. 2D). By contrast, PRL, a substrate that is largely TMEM208-independent, showed no obvious difference in release from SRP54 in the same assay (fig. S7).

Release from SRP was not affected by downstream perturbations that impair translocation. The EMC-dependent substrate TAAR5, which engages the SRP pathway (31), released from SRP54 indistinguishably when targeted to membranes containing or lacking EMC, despite a strong translocation defect in the latter (Fig. 2E). Similarly, the Sec61-dependent substrate PRL released equally well from SRP54 regardless of whether its translocation path was inhibited or not by a Sec61 inhibitor (fig. S8). This indicates that an effect on SRP release is specific to TMEM208 and not a consequence of a downstream translocation defect. We conclude that SRP release, a pre-requisite for substrates to access translocation factors, is the key step facilitated by TMEM208 for a subset of SRP-pathway clients.

## SRP54 M domain directly engages TMEM208

**Fig. 3:**
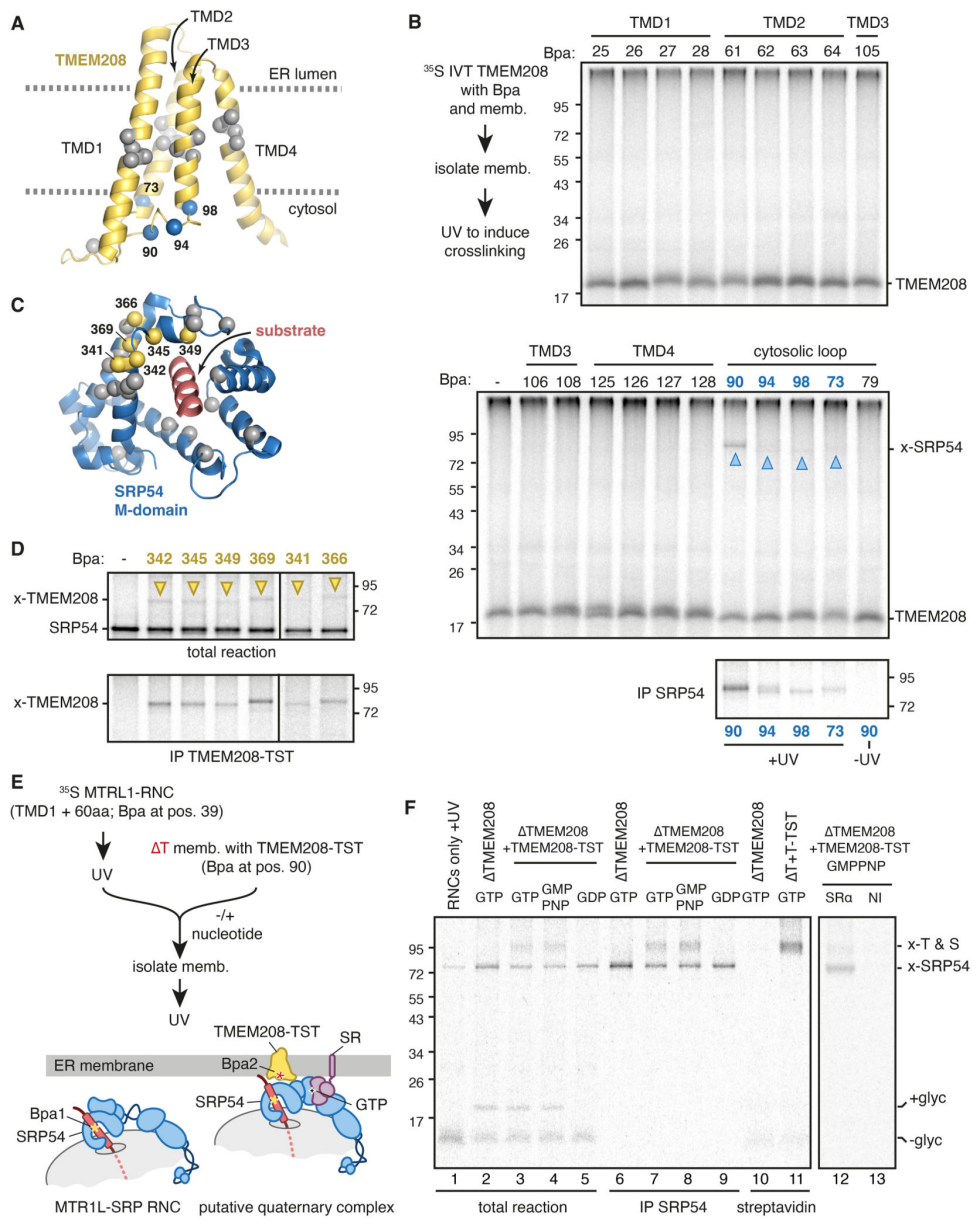
Interaction between TMEM208 and SRP54 M domain. (A) AlphaFold2 predicted structure of TMEM208 with blue C-α spheres to indicate sites that photo-crosslink to SRP54 and grey C-α spheres for sites that do not photo-crosslink. (B) Site-specific photo-crosslinking with ^35^S-methionine-labelled TMEM208 introduced by in vitro translation into ΔTMEM208 microsomes. The site of Bpa incorporation is indicated, as are the sites of SRP54 crosslinking (x-SRP54; blue triangles) as verified by anti-SRP54 IP (bottom panel). (C) Structure of SRP54 M domain (PDB: 7obr) with yellow C-α spheres to indicate sites that photo-crosslink to TMEM208 and grey C-α spheres for sites that do not photo-crosslink. (D) Site-specific photo-crosslinking with ^35^S-methionine-labelled SRP54 (containing Bpa at the indicated positions) incubated with ΔTMEM208 microsomes reconstituted by in vitro translation with TMEM208-TST (also see fig. S9). The crosslinked products were either analyzed directly (top panel) or after recovery with immobilized streptavidin to capture TMEM208-TST (bottom panel). SRP54 crosslinks to TMEM208 are indicated (x-TMEM208; yellow triangles). (E) Experimental strategy for a sequential photo-crosslinking experiment to detect a quaternary RNC-SRP54-TMEM208-SR complex. Bpa is incorporated in MTR1L at position 39 and TMEM208-TST at position 90. (F) ^35^S-methionine and Bpa-containing MTR1L-RNCs were purified through a sucrose gradient, UV irradiated to crosslink MTR1L to endogenous SRP54, then incubated with the indicated microsomes and nucleotide before a second UV irradiation. The samples of lane 1 to 11 were digested with RNase A to remove tRNA, denatured and analyzed directly or after purification via SRP54 (IP SRP54) or TMEM208-TST (streptavidin). For lanes 12 and 13, the sample was prepared as for lane 4, then solubilized by digitonin under native conditions and subjected to immunoprecipitation using anti-SRα or non-immune (NI) antibodies. Indicated are the glycosylated (+glyc), non-glycosylated (-glyc), and crosslinked products (x-SRP54, and x-T & S to denote the double-crosslinked product).

The ability to functionally complement ΔTMEM208 ER with in vitro translated TMEM208 allowed us to systematically probe its native environment using site-specific photo-crosslinking. Using the high-confidence AlphaFold2-predicted structure of TMEM208 (fig. S11) (32), we individually positioned Bpa at 20 sites encompassing each face of the four TMDs and the cytosol-facing surface (Fig. 3A,B). This wide sampling revealed a single major adduct, prominently seen from position 90 facing the cytosol, that was verified by immunoprecipitation to be SRP54. Weaker but detectable SRP54 crosslinks were seen from three other cytosol-facing positions, all of which are near M90. Thus, from among the entire ER and cytosolic proteomes, TMEM208 specifically, directly, and robustly interacts with SRP54 via a small cytosol-facing vestibule.

To identify the domain of SRP54 engaged by TMEM208, ER membranes containing functional TST-tagged TMEM208-Bpa (at position 90) were added to in vitro translation reactions of radiolabelled SRP54 or its isolated M and NG domains. Photo-crosslinking followed by purification via TMEM208-TST verified a crosslink to full length SRP54 and the isolated M domain, but not to the isolated NG domain (fig. S9A). Focusing on the M domain, we found that six of 26 Bpa-containing SRP54 variants could be photo-crosslinked to TMEM208 lacking Bpa (Fig. 3C, D; fig. S9B). As with TMEM208 crosslinking to SRP54, the sole prominent crosslink from SRP54’s M domain among all cytosolic and ER proteins was TMEM208, indicating a high degree of specificity in this interaction.

The six TMEM208-interacting positions in SRP54 (M369, Q366, L349, F345, M342 and K341) are located in two helices that contribute to the substrate binding pocket (5, 6). Notably, this side of the M domain is distal to the ribosome and would be proximal to the membrane, consistent with an ability to crosslink to TMEM208. The results show that TMEM208 engages the M domain via a subset of residues that normally face and shield the substrate. Thus, TMEM208 seems to stabilize an ‘open’ M-domain conformation where critical substrate-binding residues face TMEM208’s cytosolic vestibule. Given that there is no substrate in these experiments, the observed interaction probably reflects the post-substrate-release state of SRP54’s M domain.

To visualize the native substrate-SRP54-TMEM208 interaction, we staged a targeting reaction in which Bpa was installed in both the radiolabelled MTR1L RNC substrate and position 90 of unlabelled TMEM208-TST (Fig. 3E). Irradiation of the RNC-SRP complex generated a substrate-SRP54 adduct as expected (Fig. 3F lane 1). Addition of this crosslinked complex to ER membranes containing TMEM208-Bpa followed by UV irradiation generated an additional adduct shifted by the size of TMEM208 (lane 3). This radiolabelled product could be recovered by antibody against SRP54 and streptavidin against TMEM208-TST (lanes 7 and 11, respectively), validating a substrate-SRP-TMEM208 ternary complex.

This ternary interaction was seen with GTP (or the non-hydrolyzable analog GMP-PNP), but not with GDP, indicating that a productive interaction with SR was needed. Notably, both the substrate-SRP54 crosslink and the substrate-SRP54-TMEM208 crosslink generated with GMP-PNP could be selectively immunoprecipitated under non-denaturing conditions with antibodies against SRα (lane 12). Thus, at the time of a productive GTP-dependent (but not hydrolysis-dependent) SRP-SR interaction, SRP54’s M domain in a native targeting complex directly engages TMEM208 at the ER membrane.

## Combinatorial basis of TMEM208-dependence

Even though all SRP clients must release from SRP54 to initiate translocation, only a subset of them, primarily multipass membrane proteins, are dependent on TMEM208. We considered two potential explanations for this substrate-specificity. First, only the most tightly bound SRP54 substrates might need help from TMEM208 for release, with other substrates spontaneously dissociating fast enough to permit translocation. Second, some proteins may lose translocation competence more quickly than others, thereby increasing their dependence on prompt initiation of translocation. We found that both mechanisms can contribute to TMEM208-dependence, explaining why its substrate specificity was not easy to correlate with any single parameter.

**Fig. 4:**
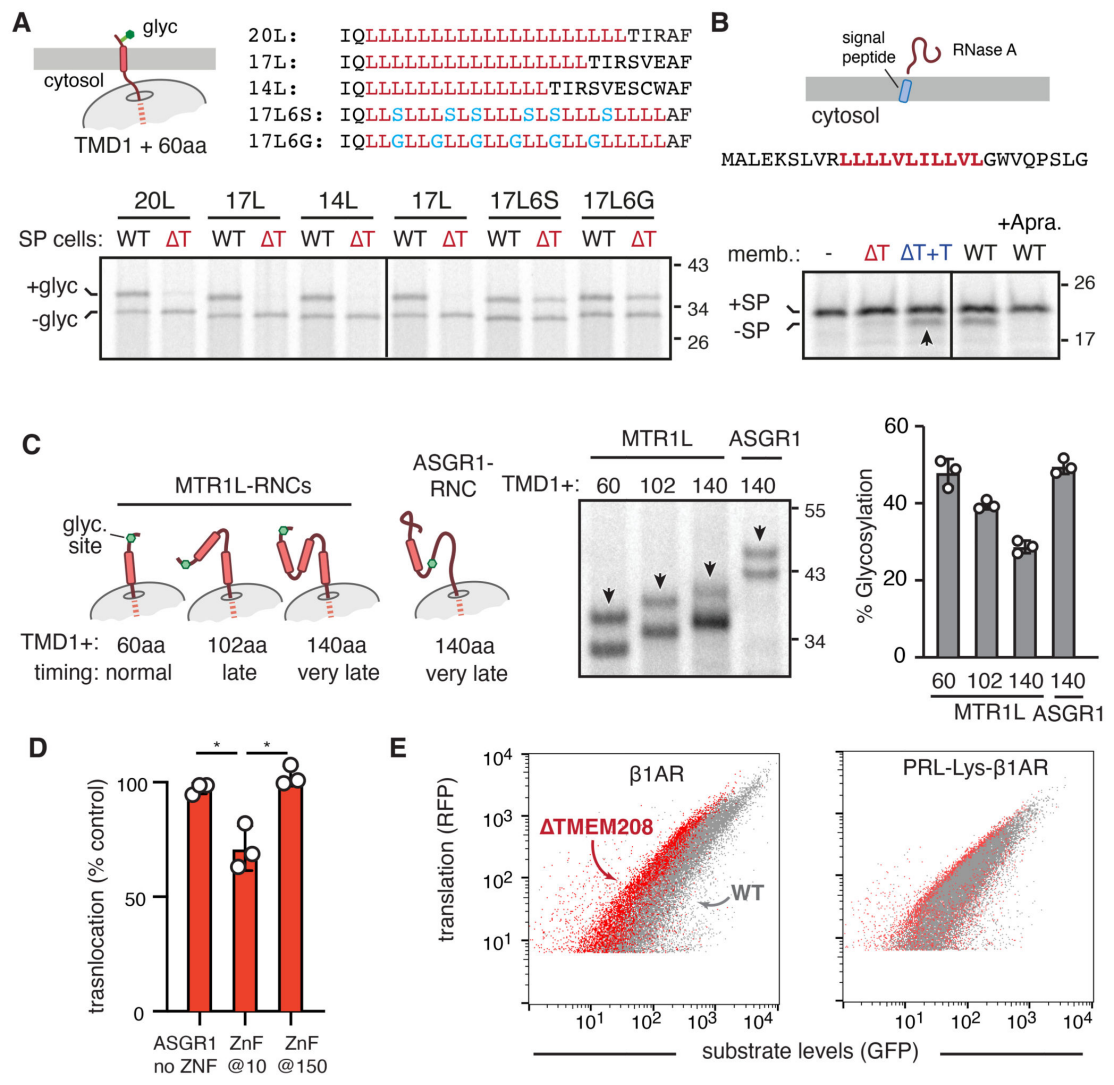
Dual-parameter basis of TMEM208-dependence. (A) Truncated ^35^S-methionine-labelled model single-TMD N_exo_ substrates (as depicted in the diagram) were translated with WT or ΔTMEM208 SP cells and monitored for insertion by glycosylation at an N-terminal site. Sequence of the TMD region of each construct is shown. (B) Diagram of RNase A and its signal peptide (with its hydrophobic core in red) are shown. ^35^S-methionine-labelled RNase A were translated in reticulocyte lysate with WT or ΔTMEM208 SP cells. Black arrows indicate the signal peptide cleaved product indicative of translocation. (C) ^35^S-methionine-labelled RNCs of MTR1L or ASGR1 of the indicated lengths downstream of the first TMD were synthesized in vitro (see diagrams on left), then incubated with microsomes. Insertion was monitored by glycosylation (arrows), which was quantified and plotted (mean ± SD, *N*=3). (D) ASGR1 lacking or containing Zn-finger (ZnF) inserted 10 or 150 amino acids downstream of the TMD was assayed for translocation into WT and ΔTMEM208 SP cells. Quantification of translocation in ΔTMEM208 SP cells relative to that seen in WT SP cells is plotted (mean ± SD, *N*=3). * p < 0.05 by paired Student's *t*-test. (E) β1AR or a variant preceded by the signal peptide of PRL and mature domain of T4 lysozyme (PRL-Lys-β1AR) was analysed by flow cytometry in WT and ΔTMEM208 cells as in Fig. 1A.

SRP54’s M domain contains a hydrophobic binding site whose size and properties would best accommodate an uninterrupted hydrophobic sequence of ∼10 residues in an alpha helix. Consistent with the idea that such a substrate should require assistance from TMEM208 for release, an artificial single-spanning membrane protein with a 20-leucine (20L) TMD was strongly TMEM208-dependent (Fig. 4A). Shortening the 20L TMD by three (17L) or six (14L) amino acids retained TMEM208-dependence. By contrast, interspersing non-hydrophobic residues into 17L to avoid long runs of leucines markedly reduced TMEM208-dependence without any impact on insertion efficiency into wild type ER.

Although many signal peptides contain a hydrophobic core of 10 residues, they are often interrupted by one or more non-hydrophobic residues (e.g., in PRL). One exception is the secretory protein RNase A, whose signal peptide contains eleven uninterrupted highly hydrophobic residues. Strikingly and unlike PRL, RNase A was strongly TMEM208-dependent (Fig. 4B). As shown in the next section, RNCs of both 14L and RNase A show strongly impaired release from SRP54 in the absence of TMEM208. Thus, an uninterrupted stretch of high-hydrophobicity seems to be one determinant of TMEM208-dependence.

To ask whether TMEM208-dependent substrates lose translocation competence more readily when their release from SRP is delayed, we mimicked delayed SRP release by using a two-stage translocation reaction. Here, we produced serially longer RNCs of MTR1L in the cytosol, then presented these substrates to wild type ER membranes (Fig. 4C). Relative to the RNC that corresponds to the timing of normal substrate release, RNCs that were 42 and 80 amino acids longer progressively lost translocation competence. By contrast, an RNC of ASGR1 comparable in length to the longest MTR1L RNC was as translocation competent as the shortest MTR1L RNC. This retention of translocation competence is similar to what was seen for secretory proteins in earlier work (33). Thus, additional polypeptide emergence from the ribosome due to a modest delay in SRP release would impact MTR1L more than secretory or single-pass proteins due to loss of translocation competence selectively of MTR1L.

To test this idea, we asked whether ASGR1 could be made partially TMEM208-dependent by causing it to lose translocation competence more quickly. Indeed, a rapid-folding Zn-finger (ZnF) domain 10 amino acids downstream of the TMD made ASGR1 partially TMEM208-dependent (Fig. 4D). By contrast, no effect on TMEM208-dependence was seen when ZnF was inserted 150 amino acids downstream of the ASGR1 TMD, a point after translocated would have already begun. Thus, prompt release of a targeting sequence from SRP54 is more important when the immediate downstream sequence is at risk of achieving a translocation-incompetent state, as would be the case for multipass membrane proteins with a series of closely spaced TMDs.

Consistent with this model, a TMEM208-dependent multipass protein (β1-adrenergic receptor) was made less TMEM208-dependent in cells simply by preceding the 7-TMD module with the PRL signal sequence followed by a soluble domain (Fig. 4E). A similar effect was seen for another GPCR, rhodopsin, when assayed in vitro (fig. S10). This indicates that multipass membrane proteins are more dependent on prompt release from SRP54 because exposure of downstream TMDs would be more likely to impede translocation than unstructured hydrophilic polypeptide. Because most single-pass and secretory proteins retain translocation competence longer, they are less reliant on prompt release. Only those with unusually hydrophobic targeting sequences, such as RNase A, are TMEM208-dependent.

## TMEM208-SRP54 interaction is functionally important

**Fig. 5:**
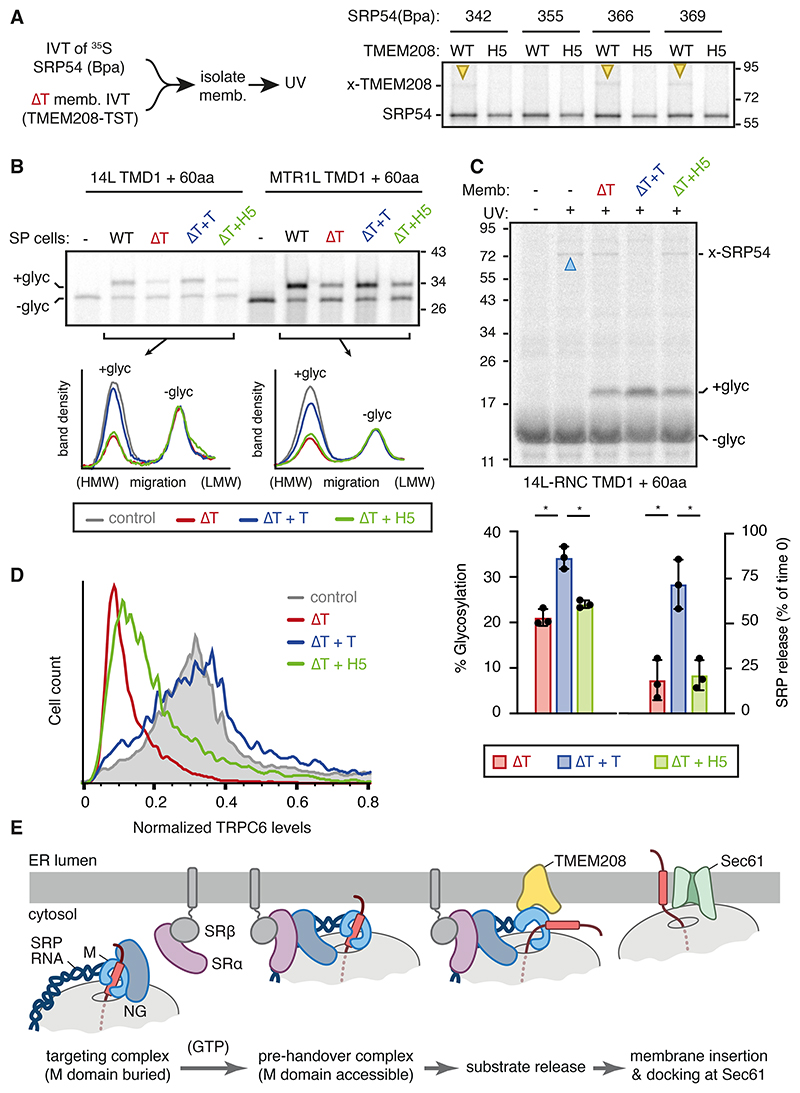
The TMEM208-SRP54 interaction is functionally important. (A) Crosslinking assay between ^35^S-methionine-labelled SRP54 and TMEM208 as in Fig. 3D. The experimental workflow is on the left. “H5” indicates mutant TMEM208 (I15S, M90S, M94S, M98S and V141S; see fig. S11), which disrupts it interaction with SRP54. (B) ^35^S-methionine-labelled 14L or MTR1L RNCs (as in Fig. 1D) were incubated with WT or ΔTMEM208 SP cells, the latter of which was first reconstituted with WT or mutant TMEM208 as indicated. Densitometry traces of each insertion reaction are shown below the gel to quantify the glycosylated (+glyc) product relative to the non-glycosylated (-glyc) product. (C) ^35^S-methionine labelled 14L RNCs were incubated for 3 min with ΔTMEM208 microsomes replenished with WT or mutant TMEM208, UV irradiated, tRNA digested and analyzed by SDS-PAGE as in Fig. 2C. Substrate glycosylation and release from SRP54 (relative to time 0) were quantified (mean ± SD, *N*=3). * p < 0.05 by paired Student's *t*-test. (D) Histogram of the dual-color TRPC6 reporter (see Fig. 1A) analysed by flow cytometry in the indicated cells. ΔTMEM208 cells were rescued by transfection with either WT or mutant TMEM208. (E) Model for the role of TMEM208 in the SRP targeting pathway.

The biological role of the TMEM208-SRP54 interaction was tested by analyzing the functional consequences of an interaction-disrupting mutation. Based on the finding that the cytosol-facing surface of TMEM208 interacts with the hydrophobic substrate-binding surface of SRP54, we mutated five cytosol-facing hydrophobic residues of TMEM208 (construct H5; see fig. S11). When reconstituted into ΔTMEM208 ER membranes in vitro or expressed in ΔTMEM208 cells, the H5 mutant of TMEM208 did not crosslink to any of several positions in SRP54 that crosslink to wild type TMEM208, despite being expressed at comparable levels (Fig. 5A; fig. S12 A-C).

The H5 mutant reconstituted into ΔTMEM208 ER membranes was deficient in accelerating substrate release from SRP and stimulating translocation of 14L, RNase A, and MTR1L (Fig. 5B,C; fig. S12D,E). When expressed in cells, H5 was largely ineffective in rescuing the biogenesis defect in ΔTMEM208 cells for TRPC6, MTR1L and TRAM2 (Fig. 5D; fig. S13). Thus, TMEM208’s interaction with the substrate-binding surface of SRP54’s M domain is crucial for its ability to accelerate cargo unloading at the ER membrane, to facilitate downstream translocation in vitro, and to facilitate membrane protein biogenesis in cells.

## Conclusions and perspective

It has long been appreciated that SRP must tightly bind and shield its hydrophobic cargo in the cytosol, then release it promptly on delivery to the ER so translocation can commence. While the basis for substrate binding and shielding has been elucidated through structural analyses (5, 6, 34–37), the mechanism of substrate release selectively at the ER was never clear. A GTP-dependent interaction between SRP and SR relocates the GTPase domains of the SRP-SR complex away from the exit tunnel in preparation for substrate release (38), but what happens next was previously obscure. Our unexpected findings on TMEM208 indicate that it is a key missing component of the pre-handover complex that directly and transiently engages the M domain of SRP54 to accelerate cargo unloading. It had presumably eluded detection because its requirement is contextual and its interaction with SRP54 is transient and sensitive to detergent.

Placing our findings in the context of earlier work, we propose the following model (Fig. 5E; fig. S14). After the initial contact between the GTPase domains of SRP and SRα, this module re-locates away from the exit tunnel to a distal site on SRP RNA (6, 9). Relocation of the GTPase domains, which requires GTP binding but not hydrolysis, exposes the previously occluded M domain, particularly the finger loop and associated helices (fig. S14). At this stage, Sec61 binding to its site on the ribosome would be precluded by the M domain still occupying the region surrounding the ribosome exit tunnel. The lifetime of this pre-handover complex is limited by the rate of GTP hydrolysis by the SRP-SR complex, which is slow in eukaryotes (10). This provides time for the M domain to engage the relatively low-abundance TMEM208 at a cytosol-facing membrane-embedded vestibule.

The TMEM208 interaction would then operate by one of two potential mechanisms. It might directly trigger a conformation change in the M domain to destabilizes the claw-like binding pocket, thereby actively facilitating release of the bound substrate. Alternatively, the M domain might dynamically open and close, with TMEM208 preferentially binding the open state to shift the equilibrium toward substrate release. Future biophysical analyses of this release step studied in isolation will be needed to determine the exact mechanism.

Once released, the hydrophobic substrate can then bind to the surface of the nearby membrane, a highly favorable and rapid reaction analogous to how amphipathic helices bind membrane surfaces (39, 40). The nascent chain is then freed to engage either EMC or Sec61, depending on its features, to initiate translocation (14, 31, 41). The SRP-SR complex, having hydrolyzed their GTP molecules by then, can dissociate from each other and the ribosome, which is then free to dock on Sec61 and finish elongation. In the absence of TMEM208, these events can still occur, albeit more slowly and at higher risk of losing translocation competence. Although SRP might slightly slow translation (42) and provide time for spontaneous substrate dissociation, this effect is modest and seems to be insufficient for multipass membrane proteins. Thus, TMEM208 is a new component of the eukaryotic SRP pathway that ensures an orderly and efficient transition from nascent chain targeting to translocation, a reaction that is crucial for many multipass membrane proteins.

## Supplementary Material

Supp table 2

Supplementary Materials

## Data Availability

All data are available in the main text or supplementary materials. Reagents used in this study are available from R.S.H. upon request.
