# Supplementary material for: Identification of a factor that accelerates substrate release from SRP
Source: Science. Author manuscript; Available in PMC 2025 Jan 24. (PMC7617331; doi:10.1126/science.adp0787)
Supplement: Supplementary Materials [file EMS202361-supplement-Supplementary_Materials.pdf]

Science
AAAS

Supplementary Materials for

Identification of a factor that accelerates substrate release from SRP

Huping Wang and Ramanujan S. Hegde

Correspondence to: rhegde@mrc-lmb.cam.ac.uk

This PDF file includes:

Materials and Methods
Figs. S1 to S14
Table S1

Other Supplementary Materials for this manuscript include the following:

MDAR Reproducibility Checklist
Table S2

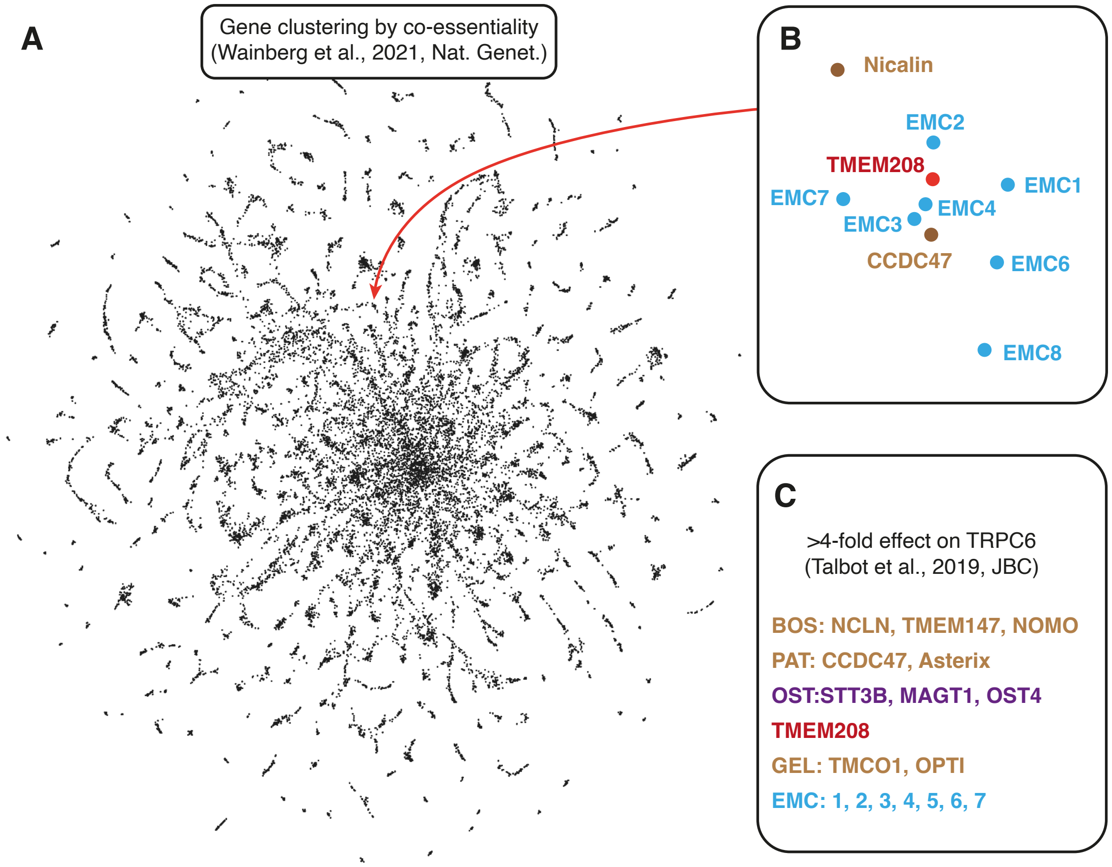

Fig. S1. Genetic interaction analysis of TMEM208.

(A, B) Co-essentiality atlas of human genes (*24*) showing that TMEM208 (red) co-clusters with genes that function in multipass membrane protein biogenesis including EMC subunits (blue) and multipass translocon (MPT) subunits (tan). (C) TMEM208 is among the list of top hits from a published genetic screen for TRCP6 surface expression (*25*). Other top hits include subunits of EMC, each of the three MPT subcomplexes, and oligosaccharyl transferase (OST).

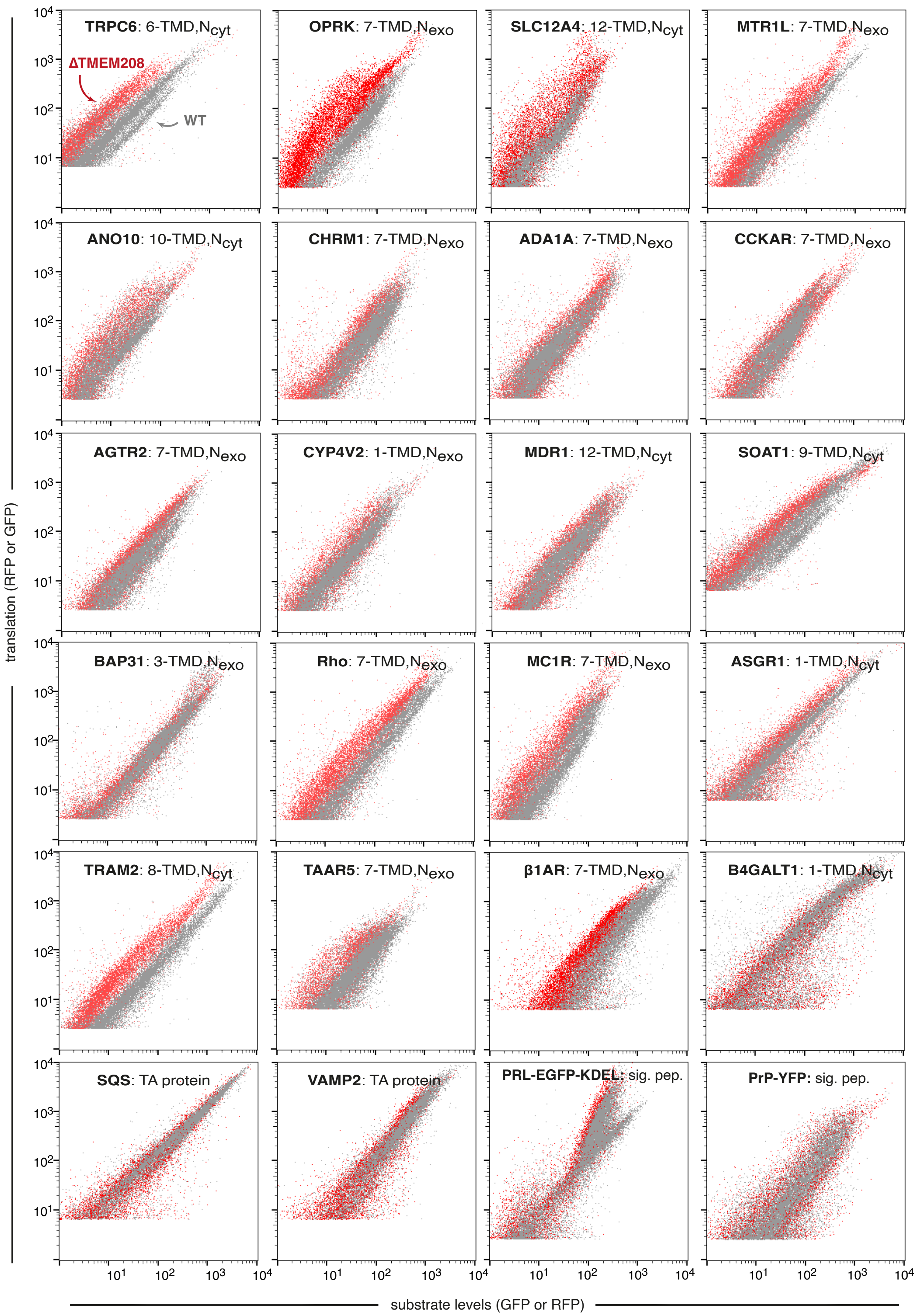

TRPC6: 6-TMD,Ncyt
ΔTMEM208
WT
OPRK: 7-TMD,Nexo
SLC12A4: 12-TMD,Ncyt
MTR1L: 7-TMD,Nexo
ANO10: 10-TMD,Ncyt
CHRM1: 7-TMD,Nexo
ADA1A: 7-TMD,Nexo
CCKAR: 7-TMD,Nexo
AGTR2: 7-TMD,Nexo
CYP4V2: 1-TMD,Nexo
MDR1: 12-TMD,Ncyt
SOAT1: 9-TMD,Ncyt
BAP31: 3-TMD,Nexo
Rho: 7-TMD,Nexo
MC1R: 7-TMD,Nexo
ASGR1: 1-TMD,Ncyt
TRAM2: 8-TMD,Ncyt
TAAR5: 7-TMD,Nexo
β1AR: 7-TMD,Nexo
B4GALT1: 1-TMD,Ncyt
SQS: TA protein
VAMP2: TA protein
PRL-EGFP-KDEL: sig. pep.
PrP-YFP: sig. pep.
translation (RFP or GFP)
substrate levels (GFP or RFP)

Fig. S2. Flow cytometry analysis of membrane protein reporters.

Dual-color fluorescent reporters of the indicated membrane proteins were analyzed by flow cytometry in either wild type (WT) or TMEM208 knockout (ΔTMEM208) HEK293 cells (a subset of these data are shown in Fig. 1A). Each construct contains a translation control (either GFP or RFP) separated by a ribosome-skipping 2A peptide from the membrane protein reporter tagged with either RFP or GFP, respectively. A leftward shift on the x-axis indicates reporter destabilisation relative to the translation control. The key properties of each protein are indicated as appropriate, including the topology of the first TMD (N_{exo} or N_{cyt}), the total number of TMDs, tail-anchored proteins (TA), and secretory proteins preceded by a signal peptide. Additional information for each protein is in table S1.

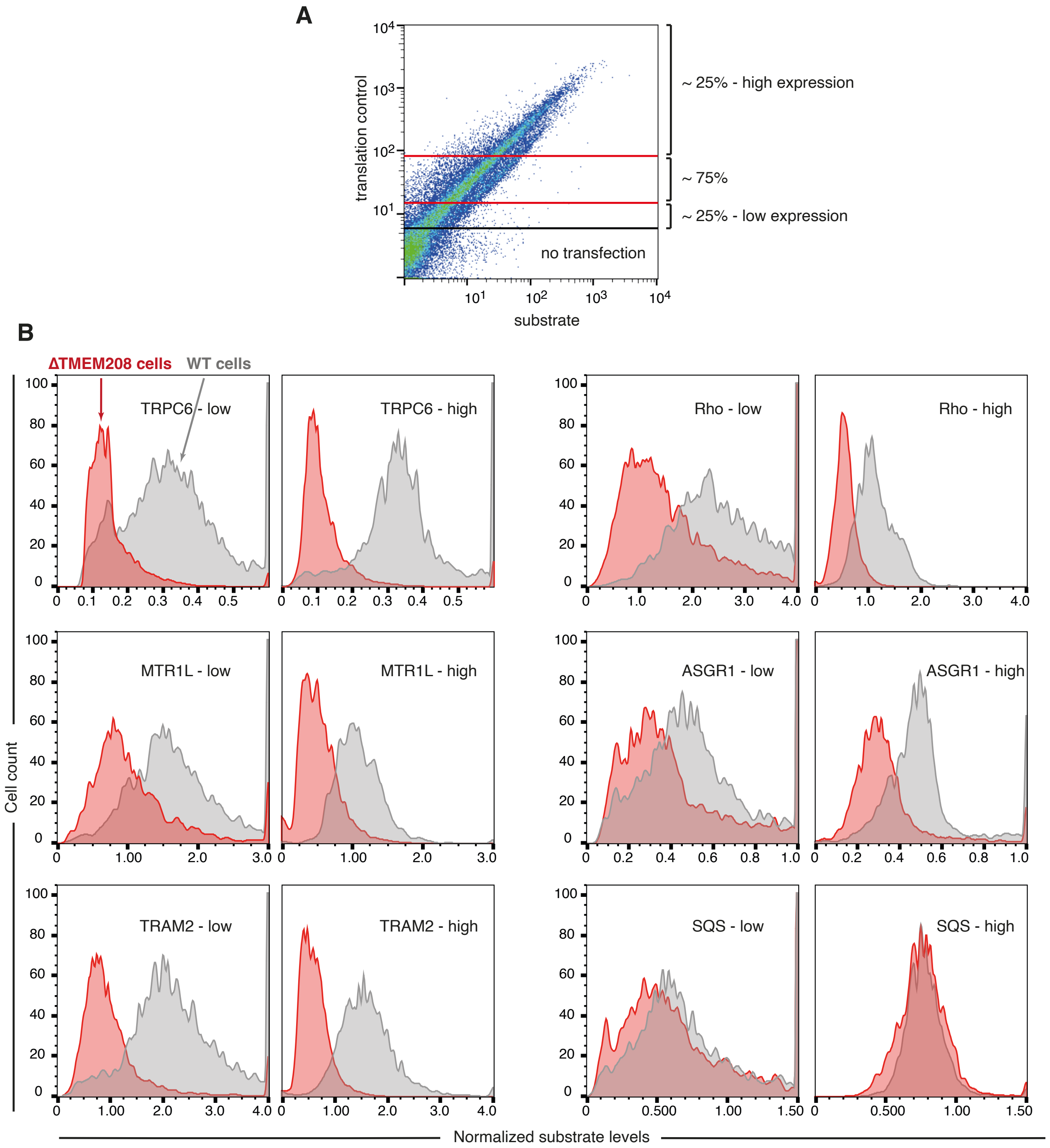

Fig. S3. Flow cytometry analysis of membrane protein reporters gated by expression.

(A) Gating strategy. The first gate excludes non-transfected cells on the basis of no reporter expression as judged by the translation control (y-axis). The second gate selects either the bottom 25% or the top 25% of the transfected cells based on expression level of the translation control. (B) Histograms of the indicated reporters analyzed by flow cytometry in the indicated cell lines as in Fig. S2 and gated as in panel A. Note that the effect (or lack of effect) of TMEM208 knockout is observed regardless of expression level.

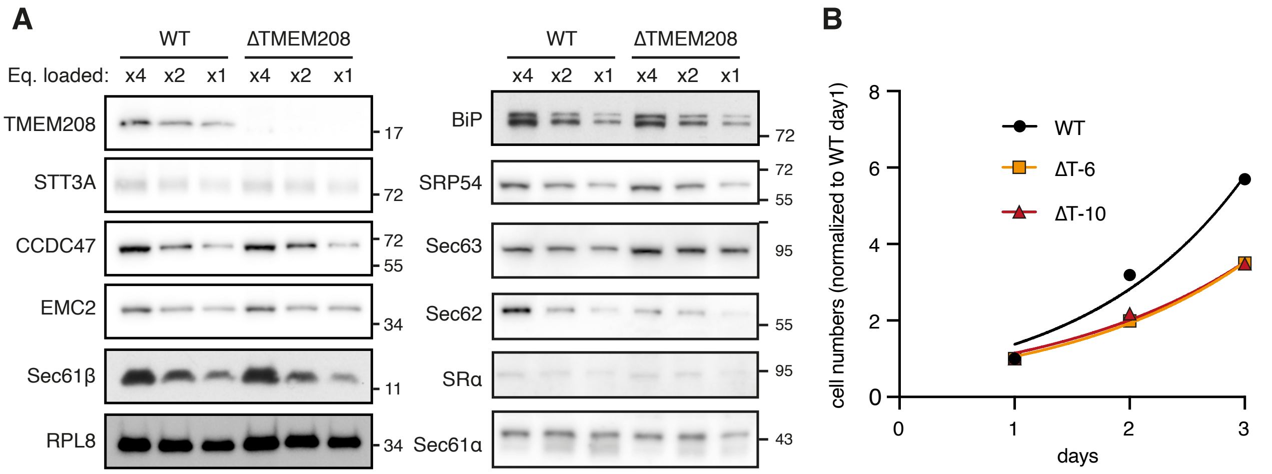

Fig. S4. Additional analysis of TMEM208 knockout cells.

(A) Serial dilutions of total cell lysate from wild type (WT) and TMEM208 knockout (ΔTMEM208) cells were immunoblotted for the indicated proteins. No appreciable changes in the expression of major protein biogenesis factors were seen. (B) Two independent TMEM208 knockout cell lines show comparable growth defects compared to parental WT cells. Mean of three independent experiments is plotted.

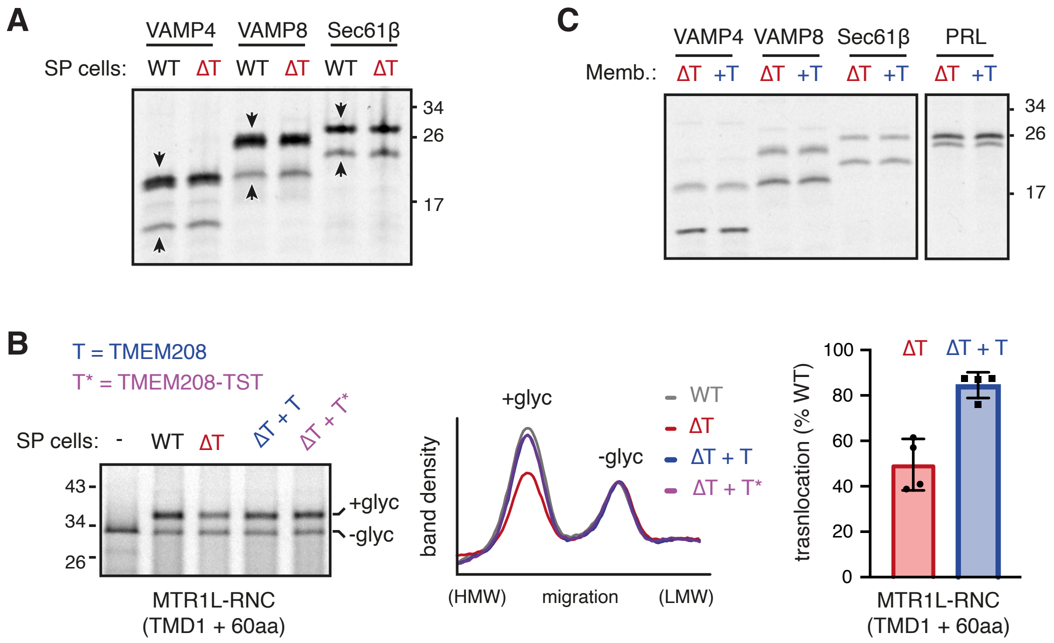

Fig. S5. Analysis of tail-anchored protein insertion into TMEM208 knockout ER.
(A) The indicated tail-anchored proteins were translated in reticulocyte lysate with ^{35}S-methionine and either WT or ΔTMEM208 (ΔT) SP cells. Black upward and downward arrows indicate the glycosylated and non-glycosylated products, respectively. (B) ΔTMEM208 SP cells were reconstituted by in vitro translation (without ^{35}S-methionine) with either WT or C-terminal TST-tagged TMEM208 (indicated by T and T*, respectively). SP cells were collected and used in an insertion reaction with ^{35}S-methionine labelled MTR1L RNCs, using WT SP cells as a positive control. Densitometry traces of each lane are shown in the middle. Substrate translocation quantified on the basis of glycosylation from multiple experiments was plotted on the right. (C) The indicated ^{35}S-methionine labelled proteins were translated in ΔTMEM208 microsomes that had first been reconstituted (or not) with unlabelled WT TMEM208 by in vitro translation. Note that no obvious effect on insertion of any tail-anchored protein was seen by either knocking out TMEM208 or by replenishing it into knockout microsomes.

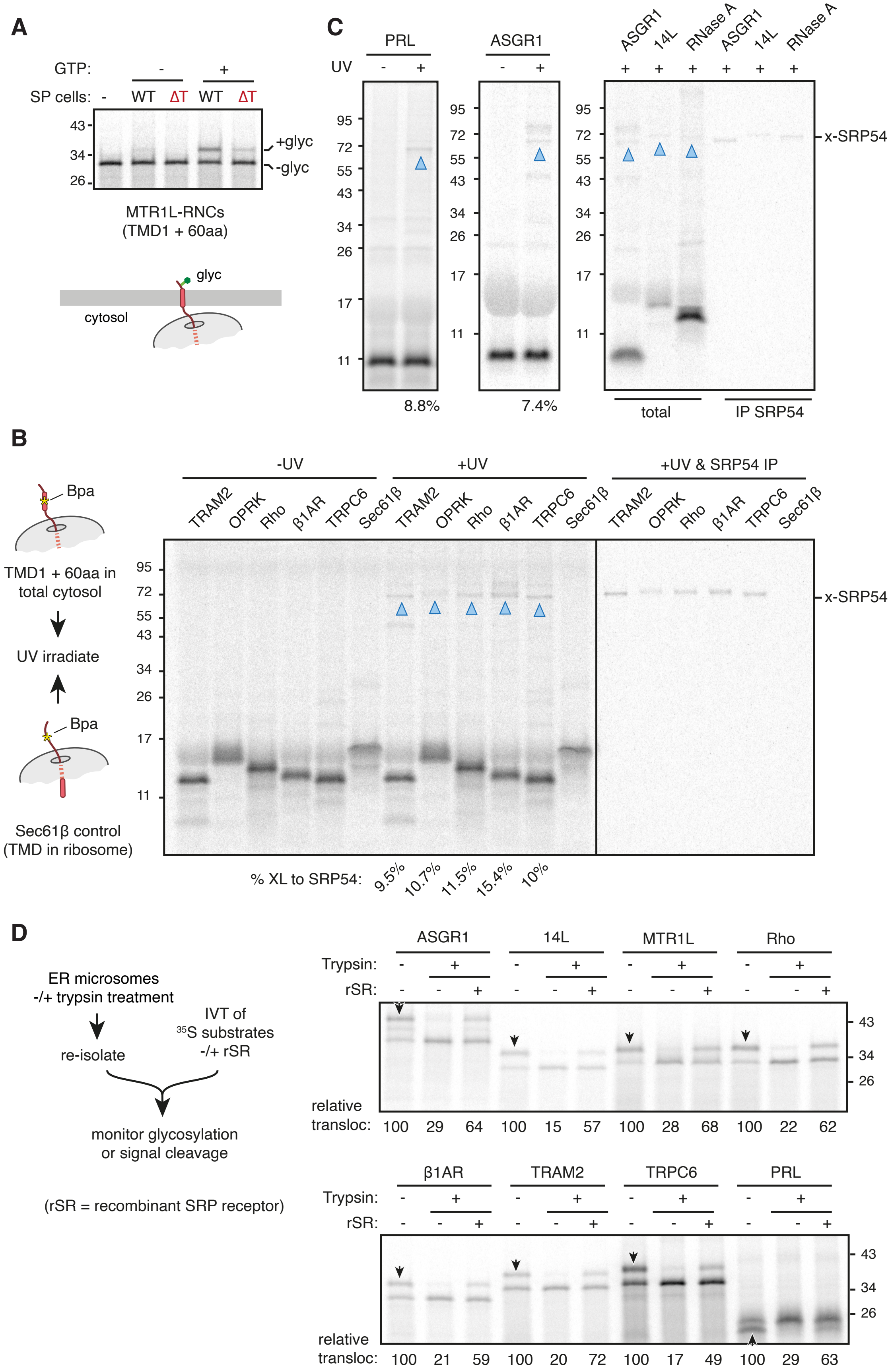
A
GTP: - +
SP cells: - WT ΔT WT ΔT
43
34
26
+glyc
-glyc
MTR1L-RNCs
(TMD1 + 60aa)
glyc
cytosol
C
PRL
ASGR1
UV - + - +
ASGR1 14L RNase A ASGR1 14L RNase A
95 72 55 43 34 26 17 11
x-SRP54
8.8%
7.4%
total
IP SRP54
B
Bpa
TMD1 + 60aa in
total cytosol
UV irradiate
Bpa
Sec61β control
(TMD in ribosome)
-UV
+UV
+UV & SRP54 IP
TRAM2 OPRK Rho β1AR TRPC6 Sec61β
x-SRP54
% XL to SRP54: 9.5% 10.7% 11.5% 15.4% 10%
D
ER microsomes
-/+ trypsin treatment
re-isolate
IVT of
35S substrates
-/+ rSR
monitor glycosylation
or signal cleavage
(rSR = recombinant SRP receptor)
ASGR1 14L MTR1L Rho
Trypsin:
rSR:
relative
transloc: 100 29 64 100 15 57 100 28 68 100 22 62
β1AR TRAM2 TRPC6 PRL
relative
transloc: 100 21 59 100 20 72 100 17 49 100 29 63

Fig. S6. Substrates use the SRP pathway for targeting.

(A) ^{35}S-methionine labelled MTR1L RNCs as in Fig. 1D were purified via sucrose gradient fractionation and tested for insertion into the indicated SP cells without or with GTP. The glycosylated (+glyc) and non-glycosylated (-glyc) products are indicated. (B, C) RNCs encoding the N-terminal region of indicated membrane proteins were produced by in vitro translation with ^{35}S-methionine and the UV-activatable crosslinking amino acid Bpa installed by amber suppression in the first TMD, or for the TA protein Sec61β in the analogous region of polypeptide protruding outside the ribosome exit tunnel (diagram on the left). Samples were UV irradiated, digested of their linked tRNA, denatured and analyzed by SDS-PAGE directly or after denaturing immunoprecipitation (IP) with anti-SRP54 as in Fig. 2A. Blue triangle indicates the major crosslink, identified by IP to be SRP54 (x-SRP54). The percentage of total translation product in the SRP54 crosslinked band was quantified and shown for each construct. (D) ^{35}S-methionine labelled RNCs as indicated were incubated with ER microsomes pre-treated with or without trypsin to digest the soluble domain of SR. The insertion reaction with trypsin-treated microsomes either contained or lacked purified recombinant SRP receptor (rSR). Percentage of relative translocation as monitored by glycosylation (downward arrows) or signal cleavage (upward arrows) was quantified and is shown below the gels (the condition with untreated microsomes was set to 100%).

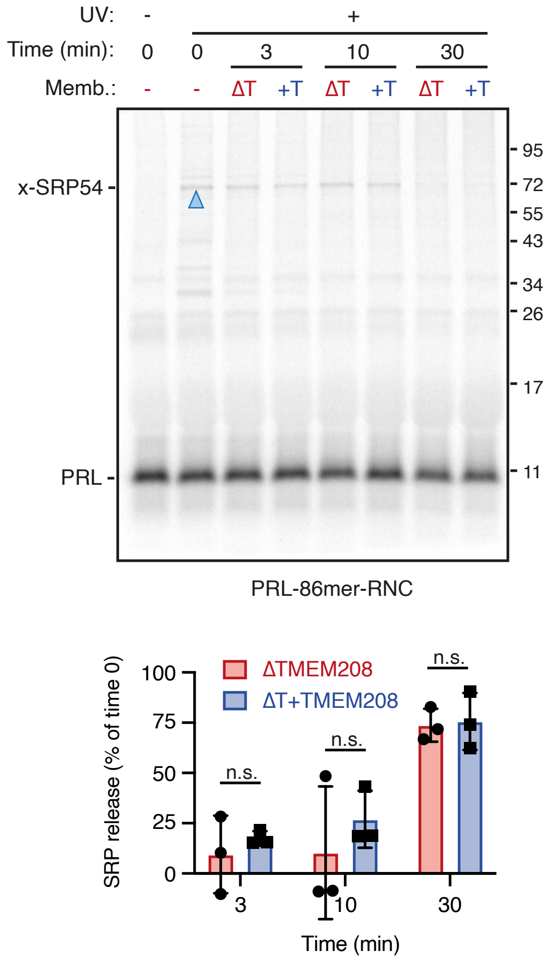

Fig. S7. Release of PRL from SRP54 is not affected by TMEM208 KO.
^{35}S-methionine labelled PRL RNCs were incubated with microsomes from ΔTMEM208 cells replenished with or without in vitro translated TMEM208 for the indicated number of minutes before UV irradiation. Substrate release from SRP54 was quantified relative to that observed at time 0. Mean ± SD of three independent experiments is plotted. n.s. $p > 0.05$ by Student's paired *t*-test. Note that due to a relatively high level of translation of the PRL construct, the number of targeting sites on the ER is comparatively limited, causing the RNCs to queue for targeting. This is the reason release appears to be slower than seen for other RNCs such as MTR1L in Fig. 2B and 2C.

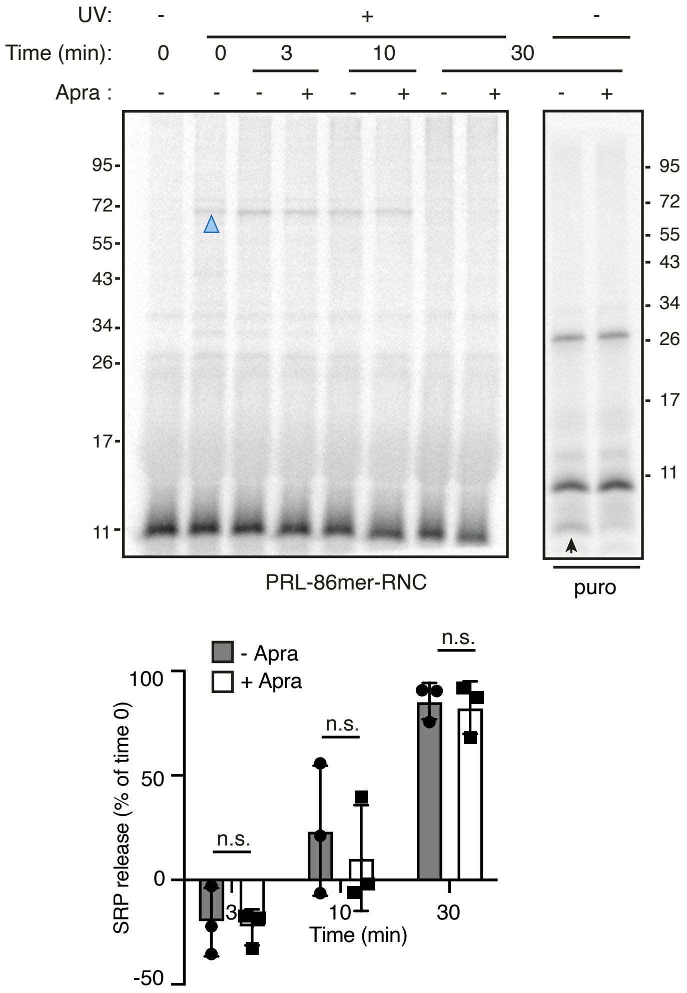

Fig. S8. Release of PRL from SRP54 is not affected by Sec61 inhibition.

^{35}S-methionine labelled PRL RNCs were incubated with microsomes from WT cells in the presence or absence of Sec61 inhibitor apratoxin (Apra) for the indicated times before UV irradiation. Substrate release from SRP54 were quantified and plotted as in fig. S7, with n.s. indicating $p > 0.05$ by Student's paired *t*-test. An aliquot of the sample at the 30 min time point was treated with puromycin to (partially) release the RNC and permit translocation (right panel). Note that the sample without Apra shows signal cleavage (upward arrow), a reaction that is substantially impaired by Apra. The puromycin reaction is incomplete as evidence by the tRNA-linked product at ~28 kD.

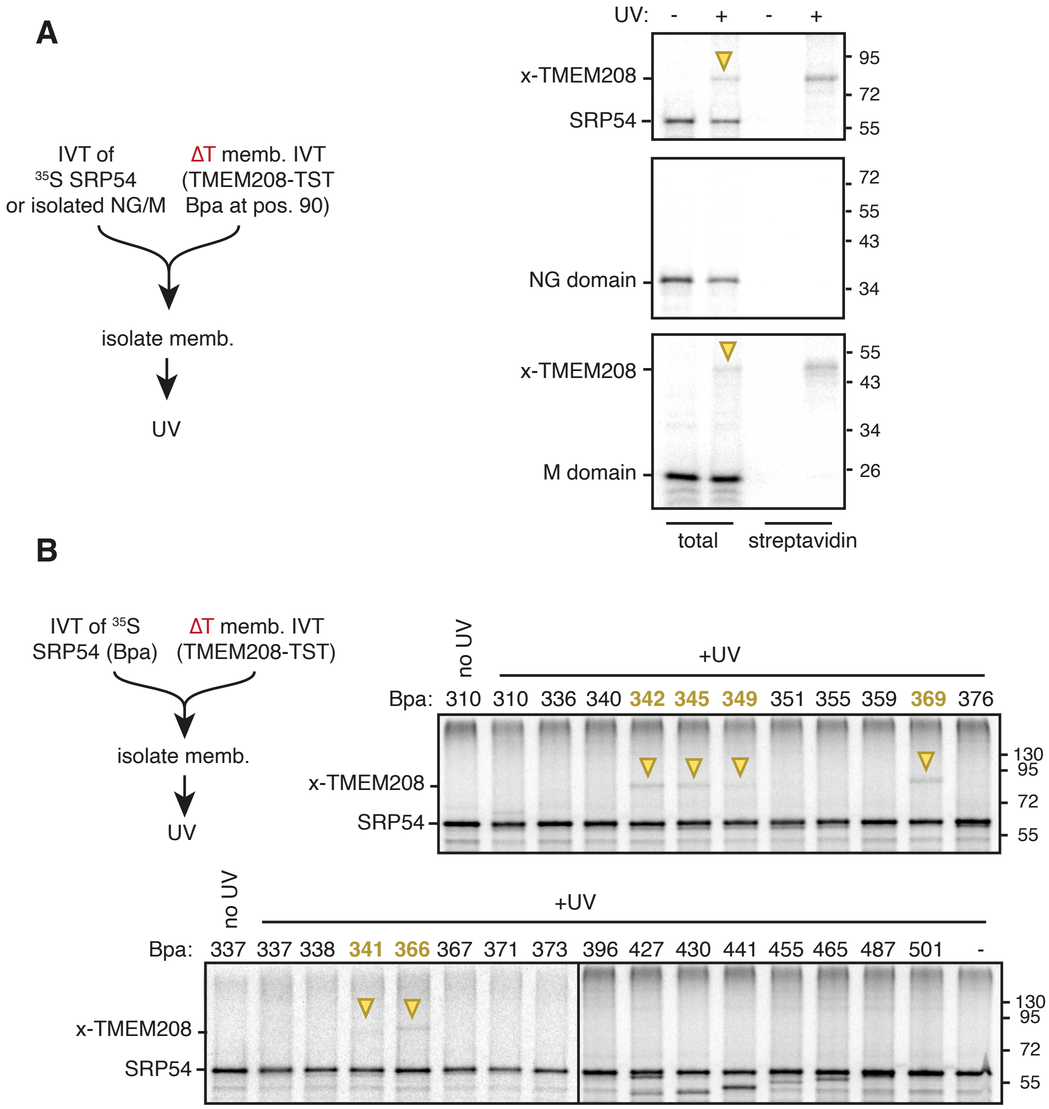

Fig. S9. TMEM208 interacts with SRP54 M domain.

(A) As diagrammed on the left, ΔTMEM208 microsomes were first reconstituted by in vitro translation with TMEM208-TST containing Bpa at position 90. Separate in vitro translation reactions were used to generate ^{35}S-methionine-labelled SRP54 or the isolated M or NG domain. After incubation of the microsomes with the SRP54 products, the microsomes were recovered by sedimentation, UV irradiated, and analyzed directly (total) or after purification of TMEM208-TST via immobilized streptavidin. Crosslinks to TMEM208 (x-TMEM208) are indicated with yellow triangles. (B) Site-specific photo-crosslinking assay as in panel A, but with Bpa installed in SRP54 at the indicated positions. Crosslinks to TMEM208 (x-TMEM208) are indicated with yellow triangles. Control reactions were either not irradiated or lacked an amber codon for Bpa incorporation.

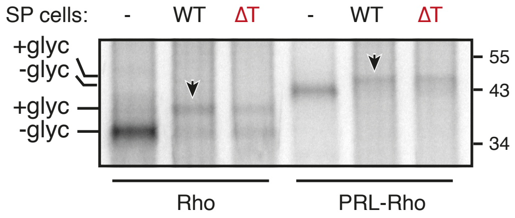

Fig. S10. Modulation of the TMEM208-dependency of Rhodopsin.

In vitro translation with ^{35}S-methionine of Rhodopsin (Rho) or a variant preceded by full length preprolactin (PRL-Rho) was carried out in the presence of WT or ΔTMEM208 SP cells. Black arrows indicate the glycosylated products. The positions of glycosylated (+glyc) and non-glycosylated (-glyc) products are indicated. Note that the effect on translocation seen for Rho with TMEM208 knockout is mostly eliminated for PRL-Rho.

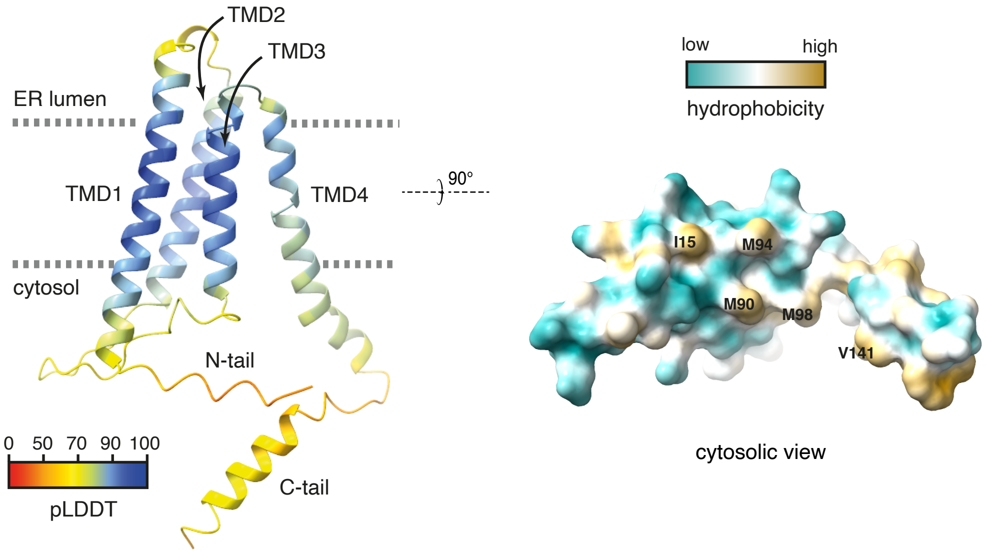

Fig. S11. Predicted structure of TMEM208.

The TMEM208 structure as predicted by AlphaFold2 (*32*) is shown colored by pLDDT (local difference distance test) or surface hydrophobicity. The structure was turned 180º to show the cytosolic surface. The five hydrophobic residues mutated to serine to generate a non-functional TMEM208 are indicated.

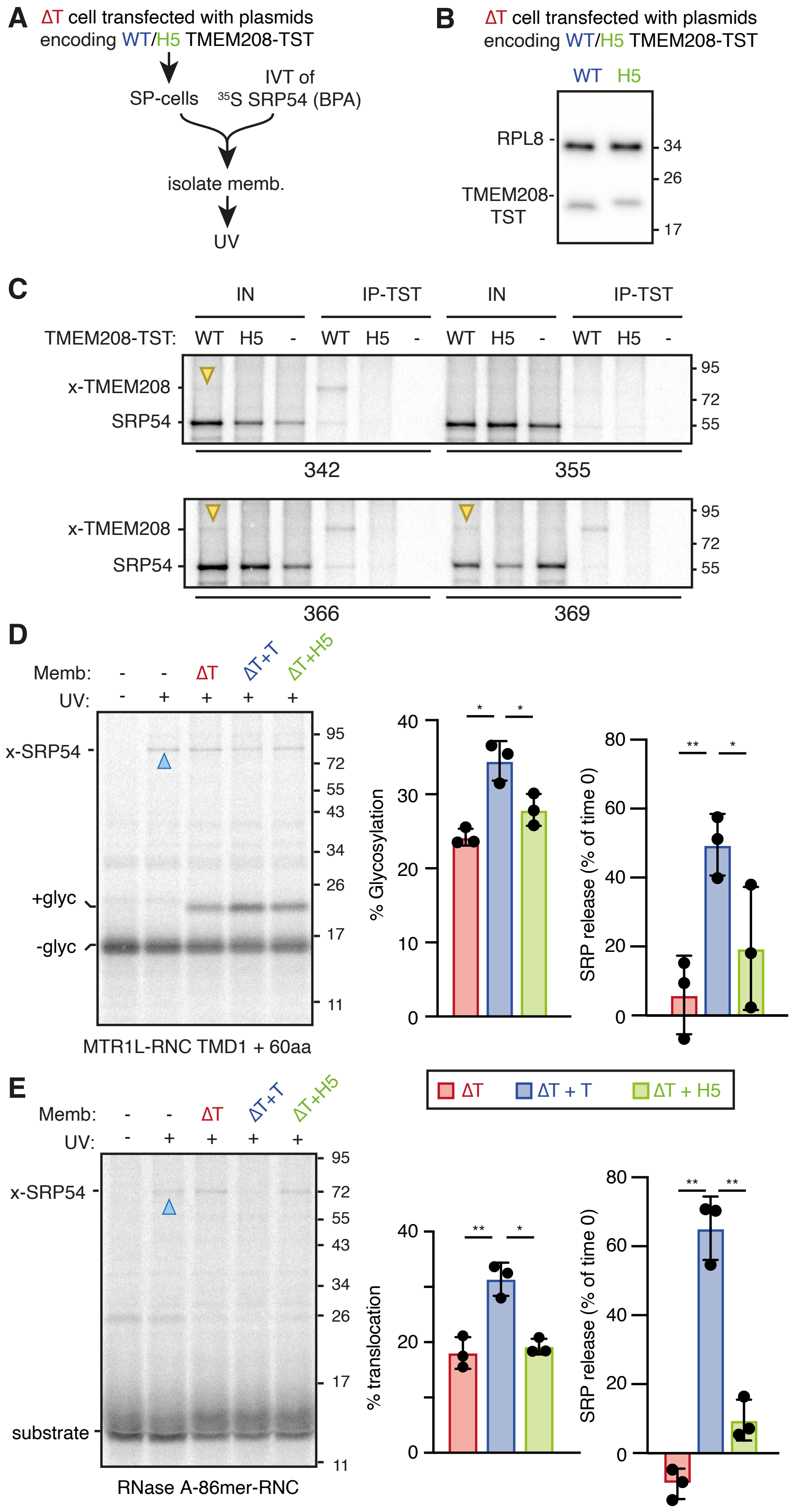

A ΔT cell transfected with plasmids encoding WT/H5 TMEM208-TST
SP-cells
IVT of 35S SRP54 (BPA)
isolate memb.
UV
B ΔT cell transfected with plasmids encoding WT/H5 TMEM208-TST
WT H5
RPL8
TMEM208-TST
34
26
17
C
IN
IP-TST
IN
IP-TST
TMEM208-TST: WT H5 - WT H5 - WT H5 - WT H5 -
x-TMEM208
SRP54
95
72
55
342
355
x-TMEM208
SRP54
95
72
55
366
369
D
Memb: - - ΔT ΔT+T ΔT+H5
UV: - + + + +
x-SRP54
+glyc
-glyc
95
72
55
43
34
26
17
11
MTR1L-RNC TMD1 + 60aa
% Glycosylation
SRP release (% of time 0)
ΔT
ΔT + T
ΔT + H5
E
Memb: - - ΔT ΔT+T ΔT+H5
UV: - + + + +
x-SRP54
substrate
95
72
55
43
34
26
17
11
RNase A-86mer-RNC
% translocation
SRP release (% of time 0)

Fig. S12. Additional analysis of mutant TMEM208 in vitro.

(A) Diagram of the experimental strategy for panel C. ΔTMEM208 cells were transiently transfected with plasmid encoding WT or mutant (H5) TMEM208-TST. A control sample was transfected with empty pCDNA5 plasmid. Cells were semi-permeabilized (SP-cells) and used to analyze photo-crosslinking to in vitro translated SRP54 containing Bpa at different positions. (B) Total cell lysates after transfection with either WT or H5 mutant TMEM208 were immunoblotted for the indicated proteins. Similar expression level of WT and H5 TMEM208-TST was observed. Cells transfected in this manner were used for panel C. (C) Following the strategy outlined in panel A, samples were analyzed directly (total) or after purification of TMEM208-TST via immobilized streptavidin. Crosslinks to TMEM208 (x-TMEM208) are indicated with yellow triangles. (D, E) ^{35}S-methionine labelled MTR1L or RNase A RNCs were incubated for 3 min with ΔTMEM208 microsomes reconstituted by in vitro translation with WT or mutant TMEM208. The samples were then UV irradiated, tRNA digested and analyzed by SDS-PAGE as in Fig. 5C. Release from SRP54 relative to that seen at time 0 was quantified and plotted (mean±SD, n=3). Substrate translocation of MTR1L was quantified from the same gels as judged by glycosylation. For RNase A, separate parallel reactions of full length protein were performed and translocation assessed by signal cleavage. * $p < 0.05$, ** $p < 0.01$ by paired Student's *t*-test.

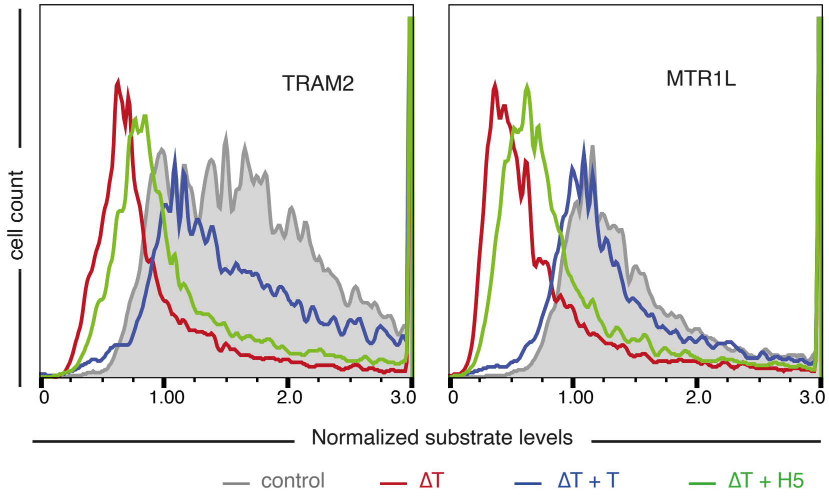

Fig. S13. Additional analysis of mutant TMEM208 in cells.

Histogram of the dual-color TRAM2 and MTR1L reporter (see fig. S2) analysed by flow cytometry in the indicated cells. ΔTMEM208 cells were rescued by re-transfection with either WT or mutant TMEM208 as in Fig. 5D.

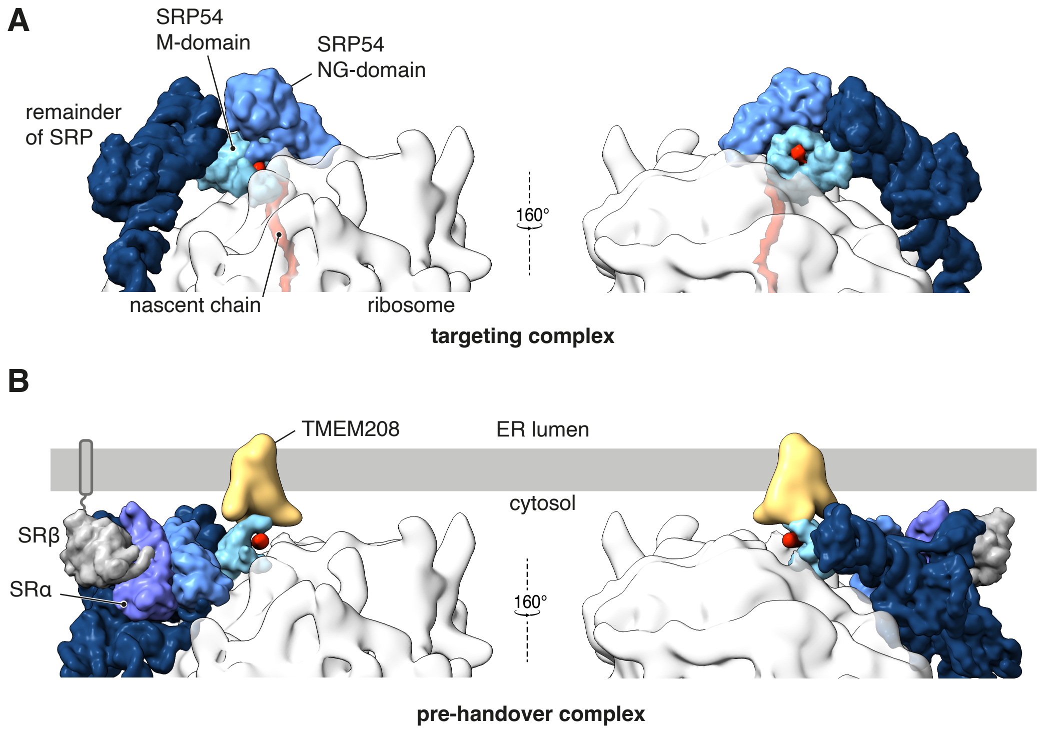

Fig. S14. Structural models of ribosome-SRP complexes.

The targeting complex and pre-handover complex [PDB: 7obr and 7obq (*6*)] are shown in panels A and B, respectively. The approximate hypothetical position of membrane-embedded TMEM208, based on site-specific photocrosslinking data, is shown in panel B. Molecular models were used to generate low-pass filtered maps (at 8 Å resolution for the factors and 20 Å resolution for the ribosome) to illustrate the relative spatial organization of SRP, SRP receptor (SR), TMEM208 and the membrane. Note that the M domain of SRP54 is inaccessible to the membrane or membrane-associated factors in the targeting complex, but becomes exposed when SRP interacts with SR and relocates the SRP54 NG domain in a GTP-dependent reaction. This pre-handover complex can now interact with TMEM208 without any clashes with the membrane.

substrate/reporter	TMEM208 dependency	Topology	Reporter position	Targeting sequence	First translocated domain length	Position of targeting sequence	ΔG of targeting sequence	Distance from targeting seq. to next TMD
OPRK	High	Nexo	C-term	VIITAVYSVVFVVGLVGNSLVMFVII	58	58	-0.399	10
TRAM2	High	Ncyto	C-term	ADIGFCLVLCVLIGLMFEVTA	32	22	-0.986	32
TRPC6	High	Ncyto	N-term	GPFMKFVAHAASFTIFLGLLVMNA	34	676	2.04	34
MC1R	High	Nexo	C-term	GLFLSLGLVSLVENALVVATIA	42	42	1.353	4
Rhodopsin	High	Nexo	C-term	FSMLAAYMFLLIMLGFPINFLTLYV	36	36	-2.58	12
SOAT1	High	Ncyto	N-term	TIYHMFIALLILFILSTLVVDYI	20	378	-2.908	20
SLC12A4	Medium	Ncyto	C-term	VYLPCLQNIFGVILFLRLTWMV	2	123	0.051	2
MTR1L	Medium	Nexo	C-term	YPPALIIFMFCAMVITIVVDLI	22	22	-1.284	20
b1AR	Medium	Nexo	C-term	SLLMALVVLLIVAGNVLVIAAIG	34	34	-1.96	12
ANO10	Medium	Ncyto	C-term	LLRRLLTSGIVIQVFPLHD	38	90	1.884	38
AGTR2	Medium	Nexo	C-term	DAIPILYYIIFVIGFLVNIVVVTLFCCQ	48	48	-2.945	5
ASGR1	Medium	Ncyto	N-term	RLLLLSLGLSLLLLVVVCVIGS	236	279	-2.628	N/A
TAAR5	Medium	Nexo	C-term	LVIYLASAAGMLIIVLGNVFV	34	34	-1.78	15
PRL-GFP-KDEL	Low	(sig. pept.)	internal	MDSKGSSQKGSRLLLLLVVSNLLLCQGVVS	247	3	0.208	N/A
CHRM1	Low	Nexo	C-term	AFIGITTGLLSLATVTGNLLVLI	25	25	0.957	13
CYP4V2	Low	Nexo	C-term	LLLWGAASALSLAGASLVLSLL	12	12	-0.682	N/A
PRL-T4L-b1AR	Low	(sig. pept.)	C-term	MDSKGSSQKGSRLLLLLVVSNLLLCQGVVS	195	0	0.208	195
ADA1A	Low	Nexo	C-term	ILLGVILGGLILFGVLGNILVIL	26	26	-2.181	23
PrP	Low	(sig. pept.)	internal	MANLGCWMLVLFVATWSDLGLC	450	3	0.34	450
CCKAR	Low	Nexo	C-term	ILLYSLIFLLSVLGNTLVITVLI	44	44	-1.717	10
MDR1	Low	Ncyto	C-term	LYMVVGTLAAIIHGAGLPLMMLV	44	48	0.599	44
B4GT1	None	Ncyto	C-term	RACRLLVAVCALHLGVTLVYYLA	256	23	-1.684	N/A
BAP31	None	Nexo	C-term	WTAVATFLYAEVFVVLLLCIPFI	5	5	-1.359	19
SQS	None	Ncyto	N-term	YSPIYLSFVMLLAALSWQYLTTL	25	237	-0.241	N/A
VAMP2	None	Ncyto	N-term	LKMMIILGVICAIILIIIIVYFT	22	238	-4.836	N/A
VAMP4	None	Ncyto	N-term	AIMALVAAILLLVIIILIVMKYG	19	117	-4.527	N/A
VAMP8	None	Ncyto	N-term	VKMIVLICVIVFIIILFIVLFAT	23	74	-5.41	N/A
Sec61b	None	Ncyto	N-term	VLVMSLLFIASVFMLHIWGKYT	19	73	-0.666	N/A

Table S1. Properties of various substrates and reporters.

Shown are the substrates used in Fig. 1 and related supplemental figures. The parameters are provded for the specific proteins analyzed (i.e., including tags and fluorescent proteins). The ΔG values are shown for either the complete targeting sequence (in the case of TMDs) or for only the hydrophobic domain (underlined, in the case of signal peptides).

Table S2. (separate file)

Plasmid and gBlock sequences introduced in this study.
